# PREP-Mt: predictive RNA editor for plant mitochondrial genes

**DOI:** 10.1186/1471-2105-6-96

**Published:** 2005-04-12

**Authors:** Jeffrey P Mower

**Affiliations:** 1 Department of Biology, Indiana University, Bloomington, IN, 47405, USA

## Abstract

**Background:**

In plants, RNA editing is a process that converts specific cytidines to uridines and uridines to cytidines in transcripts from virtually all mitochondrial protein-coding genes. There are thousands of plant mitochondrial genes in the sequence databases, but sites of RNA editing have not been determined for most. Accurate methods of RNA editing site prediction will be important in filling in this information gap and could reduce or even eliminate the need for experimental determination of editing sites for many sequences. Because RNA editing tends to increase protein conservation across species by "correcting" codons that specify unconserved amino acids, this principle can be used to predict editing sites by identifying positions where an RNA editing event would increase the conservation of a protein to homologues from other plants. PREP-Mt takes this approach to predict editing sites for any protein-coding gene in plant mitochondria.

**Results:**

To test the general applicability of the PREP-Mt methodology, RNA editing sites were predicted for 370 full-length or nearly full-length DNA sequences and then compared to the known sites of RNA editing for these sequences. Of 60,263 cytidines in this test set, PREP-Mt correctly classified 58,994 as either an edited or unedited site (accuracy = 97.9%). PREP-Mt properly identified 3,038 of the 3,698 known sites of RNA editing (sensitivity = 82.2%) and 55,956 of the 56,565 known unedited sites (specificity = 98.9%). Accuracy and sensitivity increased to 98.7% and 94.7%, respectively, after excluding the 489 silent editing sites (which have no effect on protein sequence or function) from the test set.

**Conclusion:**

These results indicate that PREP-Mt is effective at identifying C to U RNA editing sites in plant mitochondrial protein-coding genes. Thus, PREP-Mt should be useful in predicting protein sequences for use in molecular, biochemical, and phylogenetic analyses. In addition, PREP-Mt could be used to determine functionality of a mitochondrial gene or to identify particular sequences with unusual editing properties. The PREP-Mt methodology should be applicable to any system where RNA editing increases protein conservation across species.

## Background

RNA editing is a type of RNA processing (such as polyadenylation, intron splicing, and 5' end-capping) that inserts, deletes, or modifies nucleotides in an RNA transcript, thereby changing the information encoded by the genome. First discovered in trypanosome mitochondria [1], RNA editing has since been observed in a range of eukaryotes, including slime molds, amoeboid protozoans, plants, animals, and fungi, and also in viruses [2,3]. In plants, RNA editing converts cytidines to uridines and uridines to cytidines in mitochondrial and plastid, but not nuclear, transcripts. The frequency and type of conversion in each organelle is highly lineage-specific [4-7]. In angiosperms, for example, approximately 400 editing sites (all C to U) have been found in the 30 to 40 mitochondrial protein-coding genes [8-10], but only about 30 C to U sites were seen across over 100 plastid genes [11-13]. In contrast, both types of conversion are found with high abundance in mitochondrial and plastid transcripts of ferns and hornworts [4-6,14,15].

In all plant lineages, RNA editing most often alters the amino acid sequence encoded by protein-coding genes, but it can occasionally generate new start codons, create or remove stop codons, or cause silent changes that do not affect the protein sequence. RNA editing has also been observed in tRNA genes, untranslated regions, and introns [2], although the frequency of editing in these regions appears to be much lower. One feature observed immediately upon the discovery of RNA editing in plants is that edited transcripts code for proteins that are more conserved across species than proteins predicted from genomic DNA [16-18]. In fact, this tendency for codon "correction" was one of the clues that led to the discovery of RNA editing, since proteins predicted from early plant mitochondrial DNA sequences contained biochemically distinct amino acids at positions that are otherwise conserved throughout eukaryotes [16-18]. These initial observations have been repeatedly confirmed in almost all subsequent studies of editing in plants, with the most notable exceptions occurring in pseudogenes [19-21], which presumably have no selective constraints on their editing sites.

Because of the changes induced by RNA editing, the protein sequences encoded by mature mitochondrial transcripts are often quite different from what is encoded by the genomic DNA. In order to correctly analyze plant mitochondrial sequences in phylogenetic, molecular, or biochemical studies, RNA editing information must be known. Experimental determination, via direct comparison of the RNA transcript sequence and genomic DNA sequence, is the *de facto *standard for identification of sites of RNA editing. Given that these experimental analyses take time and cost money, however, two general approaches have been used to predict sites of RNA editing. The first relies on the possibility that the sequence context of an edited site contains information that signals the editing machinery or associated specificity factors. Indeed, experimental analyses of the surrounding sequence context indicate that nucleotides upstream and downstream are important in specifying sites of editing [22,23]. Furthermore, over 90% of editing sites have a pyrimidine at the adjacent upstream nucleotide [8,24]. Unfortunately, attempts at identifying consensus motifs beyond this one important nucleotide have met with little success [8,24,25]. The second predictive approach exploits the tendency of RNA editing to increase protein conservation across diverse taxa. Because of this "correcting" nature of RNA editing, it is possible to scan a protein sequence alignment for unconserved amino acids. Very often, when these unconserved amino acids have the potential to be corrected by RNA editing, they are actually edited. This approach has been shown to be very successful in predicting sites of RNA editing for several genes [6,25,26], and has also been used to infer the absence of RNA editing in the entire mitochondrial genomes of *Marchantia polymorpha*, a complex thalloid liverwort, and the green algae *Chara vulgaris *and *Chaetosphaeridium globosum *[27-29]. Limited experimental evidence has so far corroborated the lack of editing in these lineages [4-6,30].

In order to test the generality of the second predictive approach for any plant mitochondrial protein-coding gene, the PREP-Mt program was designed to predict editing sites using protein sequence comparisons and the correcting nature of RNA editing. Because the test results indicate that PREP-Mt is both fast and accurate, an online tool was also developed [31]. This resource should be useful now since editing sites have been experimentally determined for only a small percentage of plant mitochondrial genes available in the sequence databases, and it will become increasingly useful as more mitochondrial genomes get sequenced in the near future. PREP-Mt may also be effective in discriminating between functional genes and pseudogenes, as well as in elucidating mechanisms of RNA editing by exposing examples of genes that do not conform to the normal editing patterns in plant mitochondria.

## Implementation

### Construction of the Aligned Sequence Database

395 full-length or nearly full-length plant mitochondrial protein-coding genes, for which RNA editing sites have been experimentally determined or from organisms (*Marchantia*, *Chara*, and *Chaetosphaeridium*) inferred to lack RNA editing capability, were collected from Genbank. Gene sequences were extracted from each file and then edited according to Genbank annotations or literature sources. The edited gene sequences were translated into proteins according to the standard genetic code. Homologous proteins were aligned using ClustalW version 1.81 [32] and manually adjusted when necessary. The Aligned Sequence Database (ASD) consisted of these protein sequence alignments (see additional files 1, 2, 3, 4, 5, 6, 7, 8, 9, 10, 11, 12, 13, 14, 15, 16, 17, 18, 19, 20, 21, 22, 23, 24, 25, 26, 27, 28, 29, 30, 31, 32, 33, 34, 35, 36, 37, 38, 39, 40, 41, 42 for alignments).

In some cases, editing site annotations in the Genbank files were associated with nucleotides that were not a cytidine. These incorrect annotations were usually the result of obvious human errors and were corrected by referring to the literature sources prior to inclusion in the database. In addition, nine sequences from *Marchantia *(*atp4, atp8, ccmFc-A, ccmFc-B, ccmFn, rpl2, rps1, rps3, rps4*), eight from *Chara *(*atp4, atp8, ccmF, rpl2, rpl5, rps1, rps3, rps4*), and seven from *Chaetosphaeridium *(*atp4, atp8, rpl2, rpl5, rps1, rps3, rps4*) aligned poorly to the other ASD sequences. Because the PREP-Mt program relies on accurate alignments to determine RNA editing sites, these divergent proteins were not included. For the same reason, non-homologous 5' and 3' extensions present in some mitochondrial proteins (e.g., *atp6 *and *rps2*) were trimmed from the alignments. After removal of the divergent sequences, 371 sequences remained in the final alignments, spanning all 42 known protein-coding genes variably present in land plant mitochondria [33]. There were 8.8 sequences in each alignment on average, with the actual numbers ranging from 22 sequences for *nad3 *to only a single *Marchantia *sequence for *rps8*.

### Algorithm for RNA editing site prediction

Given a protein-coding DNA sequence and its gene identity, PREP-Mt predicts sites of C to U RNA editing [31]. The input sequence is translated using the standard genetic code and then aligned to the homologous ASD alignment with ClustalW using default parameters and the quicktree option. Next, for each column in the protein alignment, the corresponding codon from the input DNA sequence is examined to determine whether editing is possible. If the codon contains one or more cytidines, then the set of all possible unedited and edited states for that codon is determined. For example, if the input DNA sequence contained the codon "CCG", then the set of possible states in the RNA transcript would be "CCG" (not edited), "UCG" (edited at first position), "CUG" (edited at second position), and "UUG" (edited at first and second position). The amino acid, *i*, encoded by each of the possible codon states is compared to the amino acid, *j*, from all *N *database sequences. The score for each state, *S*_*i*_, is then defined by the equation



where the match parameter, *M*_*ij*_, is determined by



**Figure 1:**
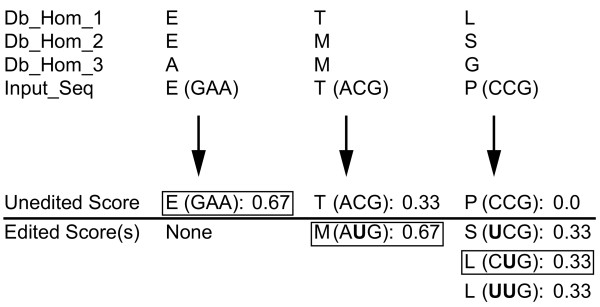
**Score calculation and prediction of RNA editing**. The hypothetical alignment shows an input sequence aligned to three homologues from the Aligned Sequence Database. The predicted state is boxed. Column 1 shows a case where RNA editing is not possible. Column 2 presents a case where the editing state is more strongly supported. Column 3 demonstrates the various rules placed on the algorithm. The CUG is taken as the correct state because it requires fewer edits than UUG and is edited at the second position, whereas UCG is edited at the first position.

Thus, the score for each possible state is a value that ranges from 0 to 1 and is simply the percentage of matches to the amino acids in the ASD sequences for that column. The state with the highest score is reported as the predicted state. In case of a tie, the state that requires the fewest number of edits is chosen as the predicted state, since the vast majority of cytidines in plant mitochondrial genes are not actually edited. Based on this rule, silent editing sites are always disfavored because, by definition, they do not affect the encoded amino acid and would therefore always tie a state that had fewer editing sites. If a tie occurs between states that require an equal number of edits, the state that is edited at the second codon position is chosen as the predicted state, since approximately 50% of all editing sites in plants occur at the second position [8,24]. An example of the scoring scheme is presented in Figure 1.

As an additional requirement, a cutoff value, *C*, can be enforced. If a cutoff value is specified, *S*_*i *_for an edited state must be greater than or equal to *C *in order to be reported as the predicted state. Thus, PREP-Mt would predict an unedited state for a particular codon if *S*_*i *_for the edited state is less than *C*, even if the edited state has a higher *S*_*i *_than the unedited state. *C *must be a value ranging from 0 to 1.

### PREP-Mt performance analyses

To evaluate the predictive performance of PREP-Mt, each sequence in the ASD was used as a test case. First, the protein sequence of the test case was removed from the ASD so that it would not be tested against itself. Then, the full-length protein-coding region for the test case was collected from its Genbank file and this unedited DNA sequence was used as input into PREP-Mt. Sites of RNA editing predicted by PREP-Mt were scored as either correct (*TP*, true positive) or incorrect (*FP*, false positive) based on comparison to the known edited sites. Similarly, sites predicted to remain unedited were scored as either correct (*TN*, true negative) or incorrect (*FN*, false negative) after comparison to the known unedited sites. This process was repeated for each sequence in the ASD, except for the single *rps8 *sequence which could not be tested because there were no other *rps8 *sequences to test against. Using the above classification, several statistical measures of predictive performance were calculated:







Because the number of known edited sites was proportionately much lower than the number of known unedited sites, the accuracy value was highly dependent on the specificity value. To determine the expected accuracy if the number of known edited and unedited sites were in equal proportion, a balanced accuracy statistic was also calculated according to the formula



Finally, to evaluate the effect of the cutoff value on the predictive performance of PREP-Mt, the performance analysis described above was rerun with *C *values ranging from 0.1 to 1.0 at all increments of 0.1.

## Results

### Classification of known editing sites

There were 60,263 cytidines present in the 370 tested protein-coding sequences (the single *rps8 *gene could not be tested because there were no *rps8 *homologues to test against). For sequences from the complete mitochondrial genomes of *Marchantia*, *Chara*, and *Chaetosphaeridium*, RNA editing capability was assumed to be absent, as indicated by predictive and experimental analyses [4-6,27-30]. For the remaining sequences, sites of RNA editing had been determined experimentally and were reported in the Genbank files and/or the literature. Based on these sources, 3,698 (6.1%) of the cytidines were classified as known sites of RNA editing, while the remaining 56,565 (93.9%) were classified as known unedited cytidines.

### PREP-Mt performance analyses

PREP-Mt was used to predict editing sites for all 370 sequences in the test set. PREP-Mt's predictive performance was measured by comparing the predicted state of each cytidine to its known state (Tables 1 and 2). Of the 3,698 known edited sites, PREP-Mt correctly identified 3,038 (*TP*) as edited sites and incorrectly predicted 660 (*FN*) as unedited sites (sensitivity = 82.2%). PREP-Mt also correctly identified 55,956 (*TN*) out of the 56,565 known unedited sites, while incorrectly predicting 609 (*FP*) to be edited (specificity = 98.9%). Altogether, PREP-Mt correctly classified 58,994 of the 60,263 cytidines in the test sequences as either an edited or unedited position (accuracy = 97.9%, balanced accuracy = 90.5%). Of the 660 false negatives, 489 occurred at first or third codon positions that did not change the amino acid encoded by the codon. Excluding these silent editing positions, which have no effect on protein sequence or function, sensitivity increased to 94.7%, accuracy increased to 98.7%, and balanced accuracy increased to 96.8%. Specificity was unaffected by the silent site adjustment. The speed of prediction was also very fast. For each of the 370 tested sequences, editing site prediction took less than one second on a 3.2 GHz Pentium IV computer running RedHat Linux 9 with 1 GB of RAM (data not shown).

To evaluate the predictive performance of PREP-Mt in more detail, the performance results were subdivided by gene (Table 1) and by genus (Table 2). After doing so, it was apparent that specificity remained very high for all treatments, never falling below 95% for any gene or genus. Accuracy was also consistently high, with only a single case lower than 90%. In contrast, sensitivity was dependent on the gene or genus analyzed. In particular, several genes (*sdh3*, *sdh4*, *atp8*, *rpl2*, *rps1*, *rps3*, and *rps19*) and genera (*Gymnocladus*, *Nicotiana*, *Oxalis*, *Podophyllum*, and *Secale*) exhibited low sensitivity scores. In some cases, the poor sensitivity scores were due to the fact that a large portion of the known edited sites were at silent positions and therefore could not be predicted by PREP-Mt. Calculating sensitivity after excluding the silent edited sites helped to alleviate many of these low scores, and in general greatly increased sensitivity scores for most genes and genera. In addition, low sensitivity may be the result of the small sample sizes found for most of the poorly performing examples, if by chance the small set of genes or genera sampled does not conform to the normal editing patterns of plant mitochondrial genes. In this regard, it is interesting to note that the editing data for *Gymnocladus*, *Oxalis*, and *Podophyllum *came from *sdh3 *and *sdh4*. It is possible that the low sensitivities seen for *Gymnocladus*, *Oxalis*, and *Podophyllum *were the result of sampling from these poorly performing genes, or, conversely, that the low scores for *sdh3 *and *sdh4 *were due to the inclusion of these poorly performing genera.

PREP-Mt predictive performance was also affected by the inclusion of sequences with unusually poor predictive results. 172 of the 609 false positives were found in genes from the three organisms (*Marchantia*, *Chara*, and *Chaetosphaeridium*) assumed to lack the ability to edit their transcripts (Table 2). The numbers of false positives across these three genomes were similar to those from the four complete genomes (*Arabidopsis*, *Beta*, *Brassica*, and *Oryza*) that do edit their transcripts, which is most likely reflective of an underlying rate of false positive prediction by PREP-Mt. However, if the assumption that *Marchantia*, *Chara*, and *Chaetosphaeridium *lack RNA editing is not correct, then some of these false positives sites may be true sites of editing. Further experimental analysis is needed to verify that RNA editing is absent in these three species. Another large portion of the false positives came from individual sequences that demonstrated poor predictive results (Table 3). Of the 58 predicted editing sites from 12 examples, 54 were false positives. On average, the false positives received moderately to very high predictive scores, making it unlikely that these examples are a collection of sequences that have a large number of spurious predictions by chance. These examples could simply be the result of incomplete experimental analysis, annotation errors, or pseudogenization. More interestingly, they may represent a small class of functional mitochondrial sequences that exhibit unusual editing properties for as yet undetermined reasons.

### Effect of the cutoff score on editing site prediction

**Figure 2:**
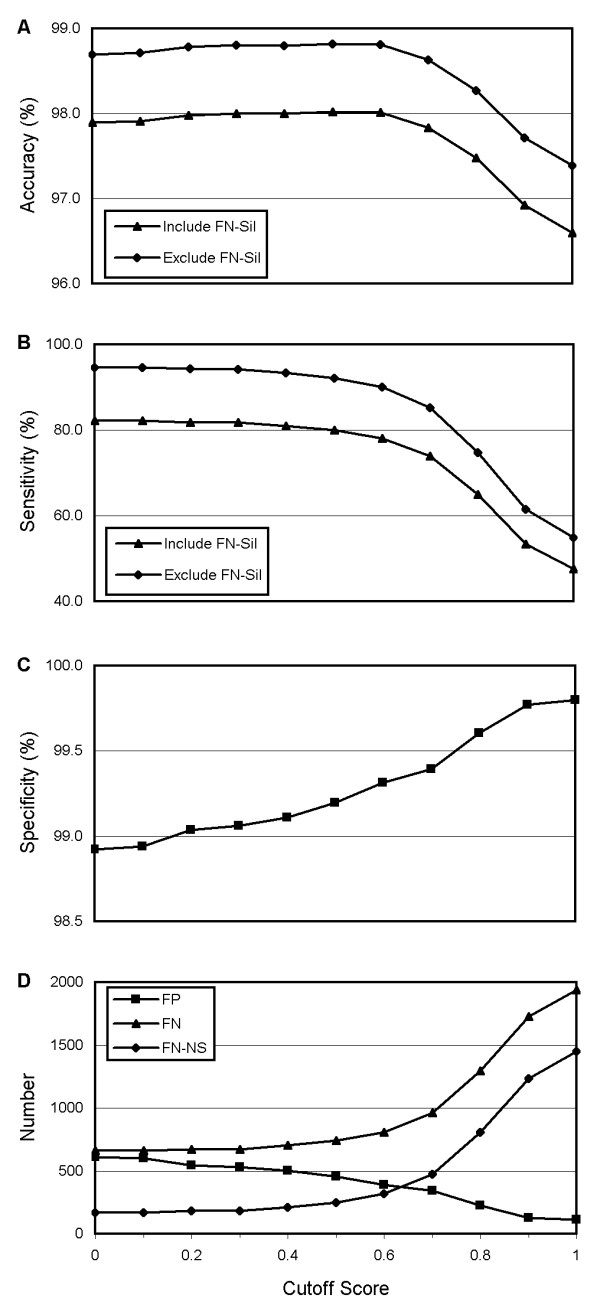
**Effect of the cutoff score on PREP-Mt predictive performance**. Effect of the cutoff score as measured by (A) accuracy, (B) sensitivity, (C) specificity, and (D) the numbers of false positives (square), false negatives (triangle), and non-silent false negatives which change the encoded amino acid (diamond). Accuracy and sensitivity were calculated using either all false negatives (triangle) or only the false negatives that change the encoded amino acid (diamond). Cutoff values used for testing ranged from 0 to 1.0 at all increments of 0.1.

The predictive analyses presented above did not impose a cutoff value (i.e., *C *= 0). To determine the effect of imposing a minimum cutoff value on editing site prediction, the predictive analyses were reevaluated using *C *values ranging from 0.1 to 1.0 (Fig. 2). As expected, increasing the cutoff value led to a decrease in the number of false positives and an increase in the number of false negatives. Up to *C *= 0.6, this was a balanced trade-off, because the number of false positives that decreased was approximately equal to the number of increased false negatives. As *C *was increased from 0.6 to 1.0, however, false negatives accumulated much faster than false positives diminished. Consequently, prediction accuracy remained roughly constant as the *C *value was increased to 0.6, but then sharply fell upon further increases of *C*. Because specificity is inversely related to the number of false positives (and is not affected by the number of false negatives), the specificity and false positive curves are also inversely related. Likewise, the sensitivity and false negative curves are inversely proportional to one another.

## Discussion

### PREP-Mt performance analysis

The PREP-Mt program is very accurate, correctly classifying 98% of all cytidines as either an edited or unedited site from 370 sequences spanning 41 functionally distinct genes and 44 diverse genera. PREP-Mt is also extremely specific and highly sensitive (at least for non-silent editing sites), properly identifying 99% of the known unedited sites and 95% of the known edited sites that change the amino acid encoded by a codon. Because these results are consistent for almost all genes and genera, the PREP-Mt methodology appears to be generally applicable for plant mitochondrial protein-coding genes. Furthermore, predictions are made exceptionally fast, which makes this program appropriate as an online resource. The speed of PREP-Mt is primarily due to the facts that the ASD homologues are prealigned, the number of sequences in each alignment is low, and mitochondrial genes are less than 1,000 nucleotides in length on average.

The high specificity of PREP-Mt is not surprising, because the methodology takes advantage of the extraordinary conservation (with rare exception) of plant mitochondrial sequences, which have the lowest known substitution rates for any organism [[34,35]; but see ref. [36]], and the limited range of amino acids possibly produced after RNA editing of a specific codon, which is usually only one out of 20. Thus, it is very unlikely that a column in a protein alignment will lead to a spurious prediction by chance. However, in poorly conserved regions of a protein alignment, one or two sequences in the ASD could by chance have the specific amino acid encoded by the edited state of an input sequence codon. As an example, initial analyses of PREP-Mt performance did not trim the non-homologous 5' and 3' extensions found in several mitochondrial proteins. Because of this, numerous sites of RNA editing were incorrectly inferred in these regions. Interestingly, the actual number of true editing sites in these same regions is extremely low, with less than one site observed on average in extensions that can span up to 1,000 nucleotides in length. Therefore, these non-homologous extensions can be removed from the ASD with almost no negative consequences.

Accuracy is also consistently high, which is due to the strong influence of specificity on the accuracy score. Accuracy is a measure of overall performance, combining the individual performances of predicting the known unedited sites (measured by specificity) and the known edited sites (measured by sensitivity). However, since the number of known unedited sites vastly outnumbers the number of known edited sites regardless of gene or genus examined, PREP-Mt's ability to identify the known unedited sites is more heavily weighted in the accuracy score. To get an unbiased measure of overall performance, sensitivity and specificity can be averaged. This balanced accuracy value represents the (biologically unrealistic) scenario in which the numbers of known unedited and edited sites are equal. Since specificity is relatively constant across all treatments, the balanced accuracy statistic correlates most strongly with the fluctuations in sensitivity.

PREP-Mt is also very sensitive in identifying the known editing sites that change the encoded amino acid, finding 95% of these non-silent editing sites. Some of the missed non-silent editing sites are likely lineage-specific gains of RNA editing that do not get predicted because the lineage is underrepresented in the ASD. Others are probably due to errors in the data that defines the known edited and unedited sites (see below). Analysis of sensitivity reveals that the main limitation of the PREP-Mt methodology is its inability to identify silent editing sites. Currently, the tie-breaking rules used by PREP-Mt always select the codon state that requires the fewest number of edits. Since any codon with the potential for silent editing produces the same amino acid regardless of editing status, the scores for the edited and unedited states will be identical and the tie-breaking rule will always select the unedited state. It would be possible to change this tie-breaking rule so that the state with the most number of edits is preferentially chosen, but doing so would lead to an overwhelming number of false positives at these potential silent editing sites, since the vast majority of these sites are not actually edited. Aligning DNA sequences instead of proteins would help to identify a number of these silent editing sites, but the chances of identifying false positives would be much greater here as well since there are only four different nucleotides in DNA as opposed to 20 amino acids in proteins. Determination of sequence motifs that unambiguously specify a particular cytidine as a site of editing would also help to predict these problematic sites. However, even if such motifs exist and are discovered, silent editing sites may remain difficult to predict. Silent editing sites are often found only occasionally edited in experimental studies, suggesting that the putative motifs are more weakly conserved for many silent editing sites.

Unlike the problem of silent editing site prediction, which is due to a real limitation of the methodology, PREP-Mt predictive performance is also negatively affected by errors in the data used to define the known edited and unedited sites, which causes some correctly predicted sites to be incorrectly classified as false positives or false negatives. In experimental determination of RNA editing sites, several types of errors are produced. In some cases, edited sites are not detected, either because a particular site is only occasionally edited or because incompletely edited transcripts are preferentially amplified during PCR. Thus, some predicted editing sites may in fact be true editing sites, but since they were not detected in the experimental analysis they are identified as false positives. In other cases, errors introduced during reverse transcription or sequencing lead to identification of C to U changes that are not actual editing sites. Inclusion of these errors in the known set of editing sites causes predicted unedited sites to be incorrectly identified as false negatives. Other noise in the known editing data comes from the fact that the Genbank annotations are subject to human error. Numerous examples of annotation mistakes were encountered while constructing the Aligned Sequence Database. Most were easily corrected because they involved a nucleotide that was not a cytidine. While three cases were known sites of the rare U to C editing in angiosperms, the vast majority were clearly mistakes. It is expected that about 25% of annotation errors would by chance happen to annotate another cytidine, and these are undetectable except by cross-referencing every known edited site with the literature. Since this was not done, a small increase in both false positives and false negatives is expected. Including sequences from *Marchantia*, *Chara*, and *Chaetosphaeridium*, which may actually perform RNA editing to some extent, and from Table 3, which may simply be the result of incomplete experimental analysis, could also cause predicted editing sites to be incorrectly classified as false positives.

### Improving predictive performance

**Figure 3:**
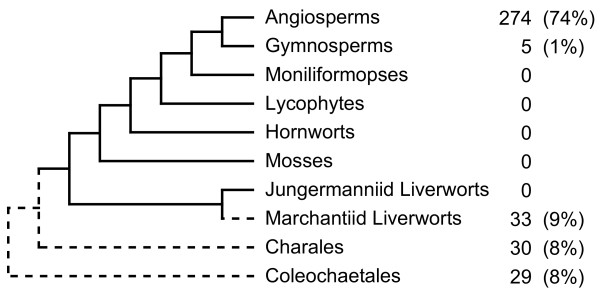
**Skewed phylogenetic distribution of sequences in the Aligned Sequence Database**. Shown are the number of sequences in the ASD for each lineage and the percentage of the total number of sequences. Editing information is available for several genes from the underrepresented groups, but they were not included in the ASD because only portions of the genes were analyzed. The dotted lines indicate lack of RNA editing based on previous predictive and experimental analyses (see main text). The marchantiid liverworts are thought to have secondarily lost the ability to perform RNA editing. The relationship between plant lineages was taken from Knoop [33].

Because the current prediction scheme calculates the score using all ASD homologues, lineage-specific gains or losses of editing may be missed due to the skewed phylogenetic distribution of sequences in the ASD (Fig. 3). Although mitochondrial RNA editing is known to occur in almost all major land plant lineages [4-6], 74% of the proteins in the ASD were from the angiosperms, while almost all of the other proteins came from organisms that most likely do not perform RNA editing. In contrast, only five sequences were available from the gymnosperms, and the other major plant lineages were not represented at all. Partial sequence data with editing information was available for a number of genes from the underrepresented groups; however, these sequences covered less than half of the gene in almost all cases and were therefore not included in the ASD.

One way to overcome the skewed sample of ASD sequences would be to modify the scoring scheme so that it approximates phylogenetic methods. Because RNA editing sites are heritable, more sites are shared between closely related species than between distantly related ones. Thus, instead of using all of the homologous ASD sequences, a subset of sequences that are closely related to the input sequence could be specified. Assigning sequence weights to the database homologues based on their overall similarity to the input sequence would achieve comparable results. The best solution to this problem would be to use actual phylogenetic methods to identify the most parsimonious or most likely state for a particular codon in the input sequence; however, the speed of prediction would certainly suffer if this strategy was implemented. An alternative approach to overcome the skewed distribution of ASD sequences would be to expand the database so that it consists of a more balanced diversity of species. This approach would also help to reduce the negative effects of small sample sizes on predictive performance. As additional mitochondrial genome and transcriptome data become available, it is expected that PREP-Mt performance will continue to increase.

The simplistic scoring scheme may also lead to spurious predictions in poorly conserved protein regions. To address this issue, a component that measures the conservation in the database sequences for each column in the alignment could be incorporated into the score. Regions of high conservation between homologues are often functionally important for that protein. Thus, the correcting nature of RNA editing is expected to be most valuable in these regions and the PREP-Mt prediction scores could be weighted based on the level of alignment conservation.

### Comparison to other prediction methods

A recent paper used sequence context and the estimated folding energy of these regions to predict editing sites in plant mitochondrial genes [24]. The authors constructed a data set containing all of the known edited sites from the complete genomes of *Arabidopsis thaliana*, *Brassica napus*, and *Oryza sativa *and compared them to an equal number of known unedited sites randomly selected from these genomes. Using tree-based statistical models, the combined data set was partitioned based on the variables that showed the greatest ability to discriminate between edited and unedited sites. Editing sites were predicted with 70.5% accuracy using a simple single-tree approach and 84.8% accuracy using a "random forest" method that analyzed thousands of trees. These values are directly comparable to the balanced accuracy of 90.5% for PREP-Mt, since the balanced accuracy statistic is the accuracy expected for PREP-Mt if an equal number of known edited and unedited sites were used. The speed of the tree-based statistical models were not presented, but they are very unlikely (especially the random forest model) to be competitive with the nearly instantaneous predictive results from PREP-Mt.

It should be noted that Cummings and Myers [24] also calculated the sensitivity and specificity of their approach; however, they classified false positives and false negatives differently than what is reported for PREP-Mt. Cummings and Myers classified false negatives as the unedited sites that were incorrectly partitioned with the true positives (the correctly classified edited sites) and false positives as the edited sites that were incorrectly partitioned with the true negatives (the correctly classified unedited sites). This has no effect on the calculation of accuracy, but it has a major effect on sensitivity and specificity scores. For their single-tree approach, Cummings and Myers provided the raw numbers of correctly and incorrectly classified edited and unedited positions, making it possible to calculate specificity and sensitivity in the same way as for PREP-Mt. This method correctly classified 1,262 out of 1,347 known editing sites (sensitivity = 93.7%), but only 637 out of 1,347 known unedited sites (specificity = 47.3%). Thus, on the one hand, their approach is very good at identifying RNA edited sites. However, approximately half of all known unedited sites are also incorrectly classified as edited sites. Because their model uses the unrealistic scenario in which the numbers of edited sites and unedited sites are equal, this problem would be increased significantly when applied to biologically realistic situations. For the 1,347 known edited sites that the authors examined from *Arabidopsis*, *Brassica*, and *Oryza*, there are actually about 17,900 cytidines that are not edited (*TN *+ *FP *from Table 2). Using their single-tree approach, over 9,000 of these unedited cytidines would be incorrectly classified as editing sites. For the random forest model, actual numbers of correctly and incorrectly classified sites were not provided, so similar calculations cannot be determined. However, because their value that incorporates the number of incorrectly partitioned unedited sites is lowest for both approaches, it seems likely that the random forest approach will also identify a substantial number of false positives in biologically realistic situations.

A second recent paper used homologous protein alignments (as does PREP-Mt) to predict C insertion editing sites in the slime mold *Physarum polycephalum *[37]. Bundschuh predicted RNA editing sites for six *Physarum *mitochondrial genes by determining their optimal states from a modified hidden markov model (HMM) that allows for cytidine insertions. Transition parameters in the HMM were defined for each gene using the position-specific scoring matrix (PSSM) from a PSI-BLAST alignment of all homologous proteins in Genbank's non-redundant database. Compared to PREP-Mt, the HMM approach was less sensitive in finding editing sites (71% on average vs. 82% for PREP-Mt) and less accurate in determining the correct amino acid sequences (92% on average vs. 99% for PREP-Mt [data not shown]). These comparisons of performance should be taken with caution since the two methods were used to predict different types of RNA editing in different organismal lineages. To allow for a more direct comparison, both approaches could be easily modified to predict the other type of editing. However, Bundschuh's use of all homologous proteins to define the PSSM may be problematic if applied to C to U editing in plant mitochondria. A significant percentage of homologues identified for a plant mitochondrial protein would be other plant mitochondrial proteins, but almost all of these sequences in Genbank are predictions based on genomic DNA. In other words, RNA editing information is not incorporated or not known and so the predicted protein sequences are not correct. Prediction of editing will get misled by inclusion of these incorrect proteins since many editing sites are shared between plants. Additionally, inclusion of non-plant homologues to define the PSSM will lead to more variable alignments, increasing the chances of spurious prediction. It will also make the beginning and end of genes more difficult to align, as already observed for Bundschuh's method which could not analyze approximately 10% of each *Physarum *gene. The PREP-Mt method gets around these problems by limiting the sequences to plants, so that the alignments are highly conserved and the protein extremities are easily aligned (except for *atp6 *and *rps2 *which have non-homologous 5' and 3' extensions for many species). Furthermore, only correct protein sequences are used in the alignments, since PREP-Mt limits the plant sequences to those with known editing information. Finally, because these alignments are predefined for PREP-Mt, Bundschuh's approach would need to use predefined HMMs for each gene to be competitive in terms of speed.

### Applications

The main use of PREP-Mt will be to identify RNA editing sites in the thousands of plant mitochondrial gene sequences available in the sequence databases, since editing information is only known for a small percentage of these sequences. Furthermore, as many additional plant mitochondrial genome sequencing projects are planned or already underway, PREP-Mt could serve an important role by quickly and accurately determining most sites of RNA editing without the need for sequencing of mitochondrial transcripts.

Currently, RNA editing information for full-length plant mitochondrial genes is limited mostly to the angiosperms (Fig. 3), which almost always convert in the C to U direction. Because of this, the reverse U to C editing phenomenon was not considered here. However, reverse editing has been found to be much more common in fern and hornwort mitochondria and was shown to increase protein conservation as well [4-6]. It is likely, therefore, that modification of PREP-Mt to allow for U to C prediction would be successful in the species that regularly perform this type of editing. Similarly, the PREP-Mt methodology could be applied to the problem of RNA editing in plant chloroplasts, which also perform editing that leads to an increase in protein conservation across species [7,11-15]. More generally, the PREP-Mt methodology should be effective for any system where RNA editing increases protein conservation across species.

PREP-Mt could also be used as a biological tool beyond simply identifying sites of RNA editing. For example, PREP-Mt could be used as a determinant of gene functionality. Unlike animals and fungi, whose mitochondrial gene content has remained stable for tens to hundreds of millions of years, gene content in plant mitochondrial genomes is much more variable [33]. The lineage-specific differences in plant mitochondrial gene content are mostly due to the proclivity of some genes to be relocated to the nuclear genome [38]. Upon transfer to the nucleus, the mitochondrial gene copy often degrades into a pseudogene. Because these pseudogenes are often still transcribed and edited [8,9,19-21], non-functionality is ascribed based on the presence of internal stop codons or frameshifts. This could be problematic since a mitochondrial gene, which has been functionally replaced by a nuclear gene, may still be intact and in-frame. Using PREP-Mt, pseudogenes could also be identified based on the fact that their editing positions do not always lead to the increased protein sequence conservation across species that functional genes demonstrate [19-21]. Intact and in-frame genes that demonstrate unusual editing properties, such as those listed in Table 3, may indicate loss of functionality and the presence of a functional nuclear copy, as already suggested for *rps1 *from *Oenothera *[39] and *rps14 *from *Brassica *[10]. It is interesting to note that 10 of the 12 cases in Table 3 are from genes that are very often found transferred to the nucleus in plants [38].

For *atp1 *and *cox3*, pseudogenization is not a likely hypothesis because these genes have never been found transferred to the nucleus in plants [38]. These two examples (as well as the 10 discussed above) could represent *bona fide *cases of functional mitochondrial transcripts that do not get edited properly for some reason. Identification of genes with abnormal editing patterns and further analysis into the causes underlying these patterns could lead to an understanding of the mechanism of RNA editing in plants, which is still largely unknown [2,3].

## Conclusion

PREP-Mt is available as an online resource that predicts sites of C to U RNA editing in plant mitochondrial protein-coding genes. It was tested on a comprehensive set of genes with known RNA editing information and was shown to be highly sensitive, specific, and accurate in most cases. The speed of prediction was also extremely fast. Thus, PREP-Mt is a substantial improvement over other RNA editing prediction methods, and its predictive performance is expected to continue to improve as more editing data become available. PREP-Mt may be useful for predicting protein sequences, for determining gene functionality, and for understanding the mechanism of RNA editing. The PREP-Mt methodology could be used to predict editing sites in any system where the effect of editing is to increase protein conservation across species, such as for reverse U to C editing in plants and for plastid RNA editing.

## Availability and requirements

PREP-Mt is an online tool that is freely available for use at 

## Supplementary Material

Additional File 1 *atp1 *alignment in FASTA format Click here for file

Additional File 2 *atp4 *alignment in FASTA format Click here for file

Additional File 3 *atp6 *alignment in FASTA format Click here for file

Additional File 4 *atp8 *alignment in FASTA format Click here for file

Additional File 5 *atp9 *alignment in FASTA format Click here for file

Additional File 6 *ccmB *alignment in FASTA format Click here for file

Additional File 7 *ccmC *alignment in FASTA format Click here for file

Additional File 8 *ccmFc *alignment in FASTA format Click here for file

Additional File 9 *ccmFn *alignment in FASTA format Click here for file

Additional File 10 *cob *alignment in FASTA format Click here for file

Additional File 11 *cox1 *alignment in FASTA format Click here for file

Additional File 12 *cox2 *alignment in FASTA format Click here for file

Additional File 13 *cox3 *alignment in FASTA format Click here for file

Additional File 14 *matR *alignment in FASTA format Click here for file

Additional File 15 *mttB *alignment in FASTA format Click here for file

Additional File 16 *nad1 *alignment in FASTA format Click here for file

Additional File 17 *nad2 *alignment in FASTA format Click here for file

Additional File 18 *nad3 *alignment in FASTA format Click here for file

Additional File 19 *nad4 *alignment in FASTA format Click here for file

Additional File 20 *nad4L *alignment in FASTA format Click here for file

Additional File 21 *nad5 *alignment in FASTA format Click here for file

Additional File 22 *nad6 *alignment in FASTA format Click here for file

Additional File 23 *nad7 *alignment in FASTA format Click here for file

Additional File 24 *nad9 *alignment in FASTA format Click here for file

Additional File 25 *rpl2 *alignment in FASTA format Click here for file

Additional File 26 *rpl5 *alignment in FASTA format Click here for file

Additional File 27 *rpl6 *alignment in FASTA format Click here for file

Additional File 28 *rpl16 *alignment in FASTA format Click here for file

Additional File 29 *rps1 *alignment in FASTA format Click here for file

Additional File 30 *rps2 *alignment in FASTA format Click here for file

Additional File 31 *rps3 *alignment in FASTA format Click here for file

Additional File 32 *rps4 *alignment in FASTA format Click here for file

Additional File 33 *rps7 *alignment in FASTA format Click here for file

Additional File 34 *rps8 *alignment in FASTA format Click here for file

Additional File 35 *rps10 *alignment in FASTA format Click here for file

Additional File 36 *rps11 *alignment in FASTA format Click here for file

Additional File 37 *rps12 *alignment in FASTA format Click here for file

Additional File 38 *rps13 *alignment in FASTA format Click here for file

Additional File 39 *rps14 *alignment in FASTA format Click here for file

Additional File 40 *rps19 *alignment in FASTA format Click here for file

Additional File 41 *sdh3 *alignment in FASTA format Click here for file

Additional File 42 *sdh4 *alignment in FASTA format Click here for file

## Figures and Tables

**Table 1:** PREP-Mt performance results subdivided by gene

Gene	No. of Species	TP	TN	FP	FN	Specificity	Sensitivity	Accuracy	Balanced Accuracy
Complex I									
*nad1*	10	135	1632	14	33 (2)	99.1	80.4 (98.5)	97.4 (99.1)	89.8 (98.8)
*nad2*	9	140	2337	22	42 (9)	99.1	76.9 (94.0)	97.5 (98.8)	88.0 (96.5)
*nad3*	22	274	1217	8	47 (4)	99.3	85.4 (98.6)	96.4 (99.2)	92.4 (99.0)
*nad4*	9	133	2358	19	12 (1)	99.2	91.7 (99.3)	98.8 (99.2)	95.5 (99.2)
*nad4L*	8	49	303	5	0 (0)	98.4	100.0 (100.0)	98.6 (98.6)	99.2 (99.2)
*nad5*	10	122	3647	29	10 (4)	99.2	92.4 (96.8)	99.0 (99.1)	95.8 (98.0)
*nad6*	10	70	995	16	15 (3)	98.4	82.4 (95.9)	97.2 (98.2)	90.4 (97.2)
*nad7*	8	117	1655	5	22 (1)	99.7	84.2 (99.2)	98.5 (99.7)	91.9 (99.4)
*nad9*	12	75	1236	7	5 (3)	99.4	93.8 (96.2)	99.1 (99.2)	96.6 (97.8)
Complex II									
*sdh3*	7	6	391	12	11 (2)	97.0	35.3 (75.0)	94.5 (96.6)	66.2 (86.0)
*sdh4*	8	10	361	14	16 (3)	96.3	38.5 (76.9)	92.5 (95.6)	67.4 (86.6)
Complex III									
*cob*	13	117	2834	19	25 (8)	99.3	82.4 (93.6)	98.5 (99.1)	90.9 (96.5)
Complex IV									
*cox1*	9	38	2936	7	7 (5)	99.8	84.4 (88.4)	99.5 (99.6)	92.1 (94.1)
*cox2*	19	193	2644	22	33 (11)	99.2	85.4 (94.6)	98.1 (98.9)	92.3 (96.9)
*cox3*	12	80	1793	21	11 (2)	98.8	87.9 (97.6)	98.3 (98.8)	93.4 (98.2)
Complex V									
*atp1*	10	21	2926	10	8 (1)	99.7	72.4 (95.5)	99.4 (99.6)	86.0 (97.6)
*atp4*	7	51	730	21	18 (8)	97.2	73.9 (86.4)	95.2 (96.4)	85.6 (91.8)
*atp6*	12	88	1893	11	10 (2)	99.4	89.8 (97.8)	99.0 (99.3)	94.6 (98.6)
*atp8*	8	16	757	10	10 (2)	98.7	61.5 (88.9)	97.5 (98.5)	80.1 (93.8)
*atp9*	19	91	792	1	17 (0)	99.9	84.3 (100.0)	98.0 (99.9)	92.1 (99.9)
Cytochrome c biogenesis									
*ccmB*	9	216	1013	14	63 (6)	98.6	77.4 (97.3)	94.1 (98.4)	88.0 (98.0)
*ccmC*	9	187	1212	24	26 (1)	98.1	87.8 (99.5)	96.5 (98.2)	92.9 (98.8)
*ccmFc*	6	84	1631	23	30 (10)	98.6	73.7 (89.4)	97.0 (98.1)	86.1 (94.0)
*ccmFn*	6	155	2249	39	38 (21)	98.3	80.3 (88.1)	96.9 (97.6)	89.3 (93.2)
Ribosomal proteins									
*rpl2*	4	2	1057	14	6 (4)	98.7	25.0 (33.3)	98.1 (98.3)	61.8 (66.0)
*rpl5*	9	45	919	14	5 (3)	98.5	90.0	98.1 (98.3)	94.2 (96.1)
*rpl6*	3	0	138	3	0 (0)	97.9	-	97.9 (97.9)	-
*rpl16*	9	33	692	6	13 (2)	99.1	71.7 (94.3)	97.4 (98.9)	85.4 (96.7)
*rps1*	3	2	295	4	5 (5)	98.7	28.6 (28.6)	97.1 (97.1)	63.6 (63.6)
*rps2*	7	25	1489	15	7 (5)	99.0	78.1 (83.3)	98.6 (98.7)	88.6 (91.2)
*rps3*	7	54	2183	19	33 (11)	99.1	62.1 (83.1)	97.7 (98.7)	80.6 (91.1)
*rps4*	6	83	1116	22	18 (6)	98.1	82.2 (93.3)	96.8 (97.7)	90.1 (95.7)
*rps7*	8	6	664	19	2 (1)	97.2	75.0 (85.7)	97.0 (97.1)	86.1 (91.5)
*rps10*	5	8	266	9	0 (0)	96.7	100.0 (100.0)	96.8 (96.8)	98.4 (98.4)
*rps11*	3	0	222	1	0 (0)	99.6	-	99.6 (99.6)	-
*rps12*	17	87	1142	13	5 (1)	98.9	94.6 (98.9)	98.6 (98.9)	96.7 (98.9)
*rps13*	10	34	554	8	5 (2)	98.6	87.2 (94.4)	97.8 (98.3)	92.9 (96.5)
*rps14*	5	2	239	7	0 (0)	97.2	100.0 (100.0)	(97.2) (97.2)	98.6 (98.6)
*rps19*	6	16	242	11	9 (3)	95.7	64.0 (84.2)	92.8 (94.9)	79.8 (89.9)
Other									
*matR*	8	73	4109	30	20 (7)	99.3	78.5 (91.3)	98.8 (99.1)	88.9 (95.3)
*mttB*	8	100	1087	41	23 (12)	96.4	81.3 (89.3)	94.9 (95.7)	88.8 (92.8)

Overall	370	3,038	55,956	609	660 (171)	98.9	82.2 (94.7)	97.9 (98.7)	90.5 (96.8)

Values after exclusion of silent editing sites are shown in parentheses. In some cases (marked with a '-'), sensitivity and balanced accuracy could not be calculated because there were no known edited sites (i.e., TP + FN = 0).

**Table 2:** PREP-Mt performance results subdivided by genus

Genus	No. of Genes	TP	TN	FP	FN	Specificity	Sensitivity	Accuracy	Balanced Accuracy
Angiosperms									
Eudicots									
*Amphicarpaea*	1	13	143	0	0 (0)	100.0	100.0 (100.0)	100.0 (100.0)	100.0 (100.0)
*Arabidopsis*	31	344	5880	58	89 (24)	99.0	79.4 (93.5)	97.7 (98.7)	89.2 (96.3)
*Beta*	30	299	5406	52	50 (11)	99.0	85.7 (96.5)	98.2 (98.9)	92.4 (97.7)
*Brassica*	32	362	5874	53	55 (15)	99.1	86.8 (96.0)	98.3 (98.9)	93.0 (97.6)
*Cologania*	1	13	138	1	1 (0)	99.3	92.9 (100.0)	98.7 (99.3)	96.1 (99.6)
*Cucumis*	2	19	332	7	0 (0)	97.9	100.0 (100.0)	98.0 (98.0)	99.0 (99.0)
*Daucus*	5	42	820	4	9 (4)	99.5	82.4 (91.3)	98.5 (99.1)	90.9 (95.4)
*Dumasia*	1	14	141	0	1 (0)	100.0	93.3 (100.0)	99.4 (100.0)	96.7 (100.0)
*Euphorbia*	1	1	53	0	0 (0)	100.0	100.0 (100.0)	100.0 (100.0)	100.0 (100.0)
*Glycine*	2	14	566	2	2 (0)	99.6	87.5 (100.0)	99.3 (99.7)	93.6 (99.8)
*Gymnocladus*	2	1	108	4	2 (1)	96.4	33.3 (50.0)	94.8 (95.6)	64.9 (73.2)
*Helianthus*	4	39	362	1	3 (1)	99.7	92.9 (97.5)	99.0 (99.5)	96.3 (98.6)
*Lactuca*	1	16	282	1	0 (0)	99.6	100.0 (100.0)	99.7 (99.7)	99.8 (99.8)
*Lespedeza*	1	13	142	0	0 (0)	100.0	100.0 (100.0)	100.0 (100.0)	100.0 (100.0)
*Lupinus*	6	68	484	3	3 (0)	99.4	95.8 (100.0)	98.9 (99.5)	97.6 (99.7)
*Lycopersicon*	4	52	550	1	21 (3)	99.8	71.2 (94.5)	96.5 (99.3)	85.5 (97.2)
*Malus*	1	8	47	0	0 (0)	100.0	100.0 (100.0)	100.0 (100.0)	100.0 (100.0)
*Nicotiana*	2	17	258	1	12 (3)	99.6	58.6 (85.0)	95.5 (98.6)	79.1 (92.3)
*Oenothera*	23	283	4395	58	95 (34)	98.7	74.9 (89.3)	96.8 (98.1)	86.8 (94.0)
*Olea*	2	11	249	0	1 (0)	100.0	91.7 (100.0)	99.6 (100.0)	95.8 (100.0)
*Oxalis*	1	2	51	0	3 (1)	100.0	40.0 (66.7)	94.6 (98.1)	70.0 (83.3)
*Petunia*	9	84	1132	8	24 (5)	99.3	77.8 (94.4)	97.4 (98.9)	88.5 (96.8)
*Pisum*	4	53	487	4	8 (0)	99.2	86.9 (100.0)	97.8 (99.3)	93.0 (99.6)
*Podophyllum*	2	3	96	5	8 (0)	95.0	27.3 (100.0)	88.4 (95.2)	61.2 (97.5)
*Raphanus*	5	31	453	3	6 (1)	99.3	83.8 (96.9)	98.2 (99.2)	91.6 (98.1)
*Solanum*	8	58	1259	16	21 (9)	98.7	73.4 (86.6)	97.3 (98.1)	86.1 (92.7)
*Vitis*	1	20	224	1	1 (0)	99.6	95.2 (100.0)	99.2 (99.6)	97.4 (99.8)
Magnoliids									
*Magnolia*	5	79	527	2	12 (3)	99.6	86.8 (96.3)	97.7 (99.2)	93.2 (98.0)
Monocots									
*Acorus*	1	8	143	2	0 (0)	98.6	100.0 (100.0)	98.7 (98.7)	99.3 (99.3)
*Allium*	2	24	131	2	0 (0)	98.5	100.0 (100.0)	98.7 (98.7)	99.2 (99.2)
*Coix*	2	23	121	0	3 (0)	100.0	88.5 (100.0)	98.0 (100.0)	94.2 (100.0)
*Elymus*	1	12	157	2	0 (0)	98.7	100.0 (100.0)	98.8 (98.8)	99.4 (99.4)
*Hordeum*	2	12	388	1	3 (1)	99.7	80.0 (92.3)	99.0 (99.5)	89.9 (96.0)
*Oryza*	34	395	5919	71	90 (28)	98.8	81.4 (93.4)	97.5 (98.5)	90.1 (96.1)
*Ruscus*	1	16	146	1	3 (2)	99.3	84.2 (88.9)	97.6 (98.2)	91.8 (94.1)
*Secale*	2	6	353	5	3 (0)	98.6	66.7 (100.0)	97.8 (98.6)	82.6 (99.3)
*Sorghum*	5	52	479	4	12 (1)	99.2	81.3 (98.1)	97.1 (99.1)	90.2 (98.6)
*Triticum*	26	360	4748	36	79 (13)	99.2	82.0 (96.5)	97.8 (99.0)	90.6 (97.9)
*Zea*	11	104	1933	16	23 (7)	99.2	81.9 (93.7)	98.1 (98.9)	90.5 (96.4)
Gymnosperms									
*Cycas*	3	35	444	7	14 (4)	98.4	71.4 (89.7)	95.8 (97.8)	84.9 (94.1)
*Pinus*	2	32	123	5	3 (0)	96.1	91.4 (100.0)	95.1 (96.9)	93.8 (98.0)
Other									
*Chaetosphaeridium*	29	0	3011	49	0 (0)	98.4	-	98.4 (98.4)	-
*Chara*	30	0	3549	72	0 (0)	98.0	-	98.0 (98.0)	-
*Marchantia*	32	0	3852	51	0 (0)	98.7	-	98.7 (98.7)	-

Overall	370	3,038	55,956	609	660 (171)	98.9	82.2 (94.7)	97.9 (98.7)	90.5 (96.8)

Notes are the same as for Table 1.

**Table 3:** Examples of unusually poor predictive results

Gene	Species	TP	FP	FN	Score
*atp1*	*Secale cereale*	0	5	1 (0)	0.96
*cox3*	*Oryza sativa*	1	13	0 (0)	0.93
*rpl2*	*Arabidopsis thaliana*	0	3	1 (0)	0.78
*rpl2*	*Brassica napus*	1	3	1 (0)	0.78
*rpl2*	*Oenothera berteriana*	1	5	3 (3)	0.67
*rpl2*	*Oryza sativa*	0	3	1 (1)	0.67
*rps1*	*Oenothera berteriana*	0	3	0 (0)	1.00
*rps7*	*Arabidopsis thaliana*	0	2	0 (0)	0.93
*rps12*	*Oryza sativa*	0	6	0 (0)	0.95
*rps14*	*Brassica napus*	0	3	0 (0)	0.67
*sdh4*	*Gymnocladus dioica*	0	4	0 (0)	0.68
*sdh4*	*Podophyllum peltatum*	1	4	4 (0)	0.68

Overall		4	54	11 (4)	0.83

Predicted score is averaged across all incorrectly predicted edited sites (FP). The numbers of FN after exclusion of silent editing sites are shown in parentheses.
